# Drug–Drug Interaction Liabilities with BTK Inhibitor TL-895

**DOI:** 10.1158/2767-9764.CRC-25-0265

**Published:** 2025-09-12

**Authors:** Jack C. Stromatt, Eman A. Ahmed, Thomas Drabison, Mahesh R. Nepal, Anika T. Chowdhury, Shelley J. Orwick, Daelynn R. Buelow, Eric D. Eisenmann, Kevin M. Huang, Alex Sparreboom, Sharyn D. Baker

**Affiliations:** 1 Division of Pharmaceutics and Pharmacology, College of Pharmacy, The Ohio State University, Columbus, Ohio.; 2 Division of Pharmaceutics and Pharmacology, Comprehensive Cancer Center, The Ohio State University, Columbus, Ohio.

## Abstract

TL-895 is an orally administered protein kinase inhibitor in clinical development for the treatment of B-cell malignancies and various other blood and autoimmune disorders. In the early stages of drug development, limited data are available to assess off-target engagement and drug–drug interaction (DDI) liabilities, which may have profound effects on drug safety and efficacy. In this context, we characterized the kinase interaction profile of TL-895 and determined that the agent inhibits Bruton’s tyrosine kinase (BTK) and bone marrow kinase on chromosome X (BMX), with more potent inhibition of BMX than BTK in a kinase assay (IC_50_: 0.53 vs. 3.02 nmol/L) and a bioluminescence resonance energy transfer (BRET) assay (IC_50_: 1.6 vs. 6.8 nmol/L). We used *in vitro* and *in vivo* models to assess DDI liabilities and identified TL-895 as a substrate of the hepatic uptake transporter OATP1B1 and the enzyme CYP3A4. *In vivo*, coadministration of TL-895 did not increase plasma concentrations of the endogenous and xenobiotic OATP1B1 substrates chenodeoxycholic acid 24-acyl-β-D-glucuronide, pravastatin, and gilteritinib, which indicates that TL-895 is an unlikely perpetrator of OATP1B1-mediated DDIs. Consistent with hepatic microsomal studies, we found that plasma concentrations of TL-895 were increased by 1.8- and 4.6-fold, respectively, in male and female mice lacking all CYP3A isoforms. The pharmacokinetic profile of TL-895 was not significantly sexually dimorphic or strain-dependent at drug doses producing human-equivalent measures of systemic exposure. These collective findings signify an important contribution of OATP1B1 and CYP3A4 to the *in vivo* handling of the dual BTK/BMX inhibitor TL-895 and suggest the agent is an unlikely perpetrator of potentially deleterious DDIs in polypharmacy regimens.

**Significance:**

TL-895 is an investigational second-generation BTK inhibitor for the treatment of B-cell malignancies. We found that TL-895 undergoes hepatocellular uptake by OATP1B-type transporters in advance of extensive CYP3A-mediated metabolism but is unlikely to perpetrate pharmacokinetic DDIs that could compromise drug safety in the context of polypharmacy regimens.

## Introduction

TL-895 (previously M7583) is an orally administered, second-generation, irreversible inhibitor of Bruton’s tyrosine kinase (BTK; ref. 1) undergoing preclinical and early-stage clinical development for B-cell malignancies (2, 3), myelofibrosis (4–7), and systemic lupus erythematosus (8). Although BTK is a target for the treatment of chronic lymphocytic leukemia (4, 9–13), the utility of currently approved irreversible BTK inhibitors such as ibrutinib, acalabrutinib, and zanubrutinib has been limited by acquired resistance and the occurrence of side effects mediated by off-target engagement of kinases, including ITK, TEC, and EGFR (12–14). Considering its claimed potency and selectivity toward BTK (8), TL-895 is a promising therapeutic option for chronic lymphocytic leukemia with the potential to improve safety over existing BTK inhibitors and induce more on-target, durable responses (15).

Despite the translational potential of TL-895, several features of small molecules that have historically resulted in clinical failure during the early stages of drug development (16–19) have not been explored for TL-895. These features include undiscovered off-target engagement leading to toxicity and pharmacokinetic drug–drug interaction (DDI) liabilities that can have costly effects on patients’ health and quality of life and induce an economic burden on the healthcare system (20). In particular, other BTK inhibitors have been characterized as substrates of CYP3A metabolism with bioavailability <3% (21), and there has been increasing recognition that hepatic uptake through OATP1B-type transporters is an important determinant for the disposition of tyrosine kinase inhibitors (TKI); however, this has been complicated by inconsistencies in the literature (22). To assist with the preclinical-to-early clinical evaluation of TL-895, we sought to (i) characterize the kinase selectivity and potency of TL-895; (ii) determine substrate and inhibitory properties toward OATP1B1-mediated transport and CYP3A4-mediated metabolism, the main elimination pathways involved in DDIs associated with protein kinase inhibitors (22, 23); and (iii) comprehensively characterize the preclinical pharmacokinetics of TL-895 and assess the *in vivo* DDI liability with OATP1B-type transporter substrates.

## Materials and Methods

See Supplementary Information, Appendix, for additional Materials and Methods.

### Chemicals and reagents

TL-895 (CID: 86721856) and rifampin (CID: 135398735) were purchased from MedChemExpress, and [^3^H]estradiol-17β-D-glucuronide (EβG; specific activity, 50.1 Ci/mmol) and [^3^H]estrone-3-sulfate (specific activity, 40–60 Ci/mmol) were obtained from American Radiolabeled Chemicals. [^3^H]CCK-8 (sulfated), a derivative of cholecystokinin, was purchased from PerkinElmer Health Sciences, and 8-Fluo-cAMP (8-FcA) was purchased from Enzo Biochem. For *in vitro* studies, TL-895 was prepared in DMSO (Sigma-Aldrich; CID: 679). Blank (drug-free) plasma was obtained from wild-type mice on a C57BL/6 background strain (Charles River Laboratories). Blank plasma was obtained from whole blood samples collected in 1.3 mL tubes containing heparin, which was then centrifuged at 1,500 × *g* for 5 minutes, and the supernatant was transferred and stored until further use, as described (24).

### Kinase profiling

**Figure 1.**
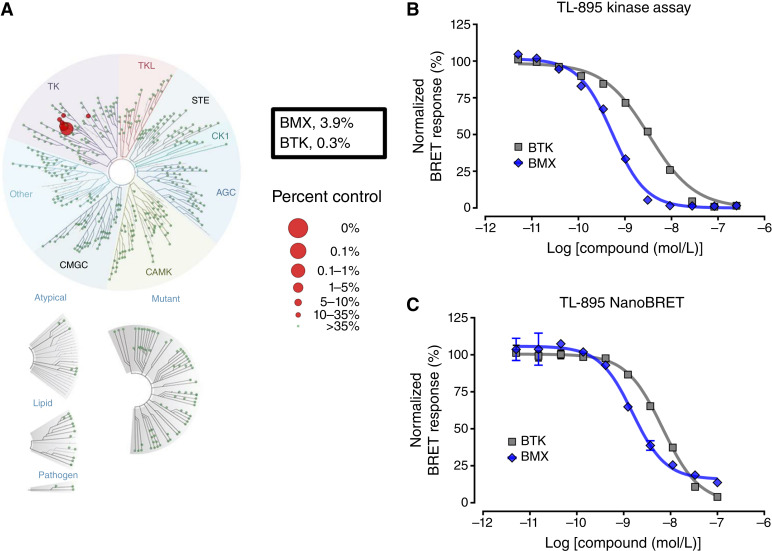
Interaction of TL-895 with protein kinases. **A,** KINOME*scan* of TL-895 (500 nmol/L) against 403 nonmutant kinases to determine a selectivity score S(35) of 0.015. **B,** Kinase profiling of TL-895 against BTK and BMX revealed EC_50_ values of 3.02 and 0.53 nmol/L, respectively. Data represent the mean values (symbols) and SD (error bars) of two independent experiments performed in duplicate. **C,** Bioluminescence resonance energy transfer (BRET) luciferase response of BTK and BMX in the presence of TL-895 in HEK293 cells expressing BTK- and BMX-NanoLuc Fusion Vector showing EC_50_ values of 6.8 and 1.6 nmol/L, respectively. Data represent mean values (symbols) and SD (error bars) of two independent experiments performed in duplicate.

The kinase selectivity for TL-895 was previously assessed using a Millipore Kinase Profiler screening panel testing 1 μmol/L against 270 kinases (8). In this study, we expanded these findings by testing the binding affinities of TL-895 (500 nmol/L) to a panel of 468 kinases, which were assessed using KINOME*scan* (Eurofins DiscoverX) and TREE*spot* to map kinase interactions. Kinases that bind the test compound are represented with red circles (Fig. 1A), and the circle size is proportional to the binding affinity. The compound binding to each kinase was normalized based on positive and negative control signals, in which %Ctrl = [(test compound signal – positive control signal)/(negative control signal – positive control signal)] × 100 and in which DMSO was used for the negative control. A selectivity score S(X) was calculated as S(X) = number of nonmutant kinases with %Ctrl < X/number of nonmutant kinases tested, and values for S(35), S(10), and S(1) were calculated. TL-895–mediated inhibition of LYN kinase was determined with the *Kd*ELECT assay (Eurofins DiscoverX).

### HotSpot Kinase Assays

The binding affinity of TL-895 to BTK and bone marrow kinase on chromosome X (BMX) was determined via the HotSpot Kinase Assay (Reaction Biology). Kinase activity is measured directly by producing a radiolabeled and phosphorylated product. Briefly, recombinant human BTK or BMX, 20 mmol/L HEPES–HCl, 10 mmol/L MgCl_2_, 1 mmol/L EGTA, 0.02% Brij 35, 0.1 mmol/L Na_3_VO_4,_ 0.02 mg/mL BSA, 2 mmol/L DTT, and 1% DMSO were combined at room temperature. Ten doses of TL-895 starting at 50 μmol/L with 3-fold dilutions, the control staurosporine (CID: 44259), or DMSO were added and incubated at 37°C for 20 minutes. Afterward, ATP and ^33^P-ATP were added in combination to reach 10 μmol/L. The reaction was incubated for 120 minutes followed by spotting the reaction mixture onto filter papers that bind the radioisotope-labeled product.

### NanoBRET intracellular binding assay

Intracellular binding was determined with a NanoBRET assay (Reaction Biology). In brief, human embryonic kidney (HEK293) cells were transfected with BMX-NanoLuc or BTK-NanoLuc Fusion Vector, and the transfected cells were treated for 60 minutes with TL-895 starting at 0.1 μmol/L, with 3-fold dilutions to achieve 10 concentrations tested in duplicate. The protein kinase inhibitors dasatinib and CTx-0293885 were used as positive controls.

### Cell lines for uptake studies

Uptake experiments were carried out with HEK293 (RRID:CVCL_0045) cells engineered to overexpress OATP1B1, OATP1B3, or an empty vector control (VC), as previously described (25). Cells were cultured in DMEM (Invitrogen) supplemented with 10% FBS. The culture medium for cells overexpressing OATP1B1 and its corresponding VC was supplemented with hygromycin B (25 mg/mL; Invitrogen) and blasticidin (37.5 mg/mL; Biovision). The medium for cells overexpressing OATP1B3 contained G418 sulfate (1,000 μg/mL; A.G. Scientific). Cell lines were authenticated by Applied Biosystems AmpFlSTR Identifiler testing with PCR amplification. All cells were used within passage 30 and verified to be *Mycoplasma*-free using the MycoAlert Mycoplasma Detection Kit (Lonza).

### Competitive counterflow transport

Drug–transporter interactions were examined as previously described (26) using HEK293 cells overexpressing OATP1B1 or OATP1B3 in 96-well plates. Once confluent, cells were washed once with warm PBS; warm DMEM containing EβG was added to each well and removed after 60 minutes to allow for saturation, and then a comixture of EβG and TL-895 was added and incubated for an additional 30 minutes. Cells were washed 3 times with cold PBS to stop any further uptake or efflux. Cells were solubilized in 100 μL of 1 N NaOH and placed on an orbital shaker at room temperature, and after 4 hours, 50 μL of 2 mol/L HCl was added. Next, the plate was mixed, and 100 μL aliquots were transferred to a scintillation vial, and total radioactivity was determined by liquid scintillation counting. Quantification of total protein content was performed with a Pierce bicinchoninic acid protein assay (Thermo Fisher Scientific). Uptake values were calculated by dividing the observed radioactivity (in disintegrations per minute) from each replicate by the absolute amount of total protein (in mg), and results were normalized to the uptake values in VC cells treated with DMSO.

### Transport inhibition assays

Inhibition of xenobiotic transport function was examined as previously described (27, 28), using 24- or 96-well tissue culture plates (Thermo Fisher Scientific) coated with poly-D-lysine that were kept at room temperature for 24 to 72 hours. Immediately prior to plating, poly-D-lysine was aspirated, and the plates were washed with sterile water. Plating media were prepared with phenol red–free DMEM supplemented with 10% FBS, 1 mmol/L GlutaMAX, and 1 mmol/L sodium pyruvate (Thermo Fisher Scientific). HEK293 cells overexpressing OATP1B1 and their corresponding VC cells were cultured in the presence of 1 μg/μL of doxycycline to induce expression of the transporter. All experiments were performed in 24- or 96-well plates in volumes of 0.5 mL or 100 μL per well, respectively, containing 0.5 × 10^6^ cells/mL. After a 24-hour incubation period, cells were washed with warm PBS and incubated with the desired concentration, ranging from 0.1 to 100 μmol/L, of TL-895 or the positive control inhibitor rifampin in phenol red–free and serum-free DMEM for 15 minutes. The drug-containing media were aspirated, and a coincubation of TL-895, rifampin, or DMSO was performed using 8-FcA (2.5 or 5 μmol/L) as a prototypical substrate for cells expressing OATP1B1 or EβG or CCK-8 (0.2 μmol/L) for cells expressing OATP1B3 using a 15-minute incubation period. The experiment was then terminated with three washes of ice-cold PBS. 8-FcA concentrations were estimated by fluorescence measurements at excitation and emission wavelengths of 485 and 535 nm, respectively, and radioactivity was measured by liquid scintillation counting. Total protein levels were determined with a bicinchoninic acid protein assay. Uptake values were calculated by dividing the fluorescence or disintegrations per minute from each replicate by the amount of total protein (mg), and results were normalized to the uptake values in VC cells treated with DMSO.

To assess the potential *in vivo* relevance of the observed IC_50_ values for transport inhibition, we utilized criteria and basic model approaches outlined previously (29), which take into consideration observed peak plasma concentrations and the fraction unbound TL-895 in plasma to derive peak concentration (C_max_)/IC_50_ ratios and R values (30). In preliminary experiments, the fraction unbound TL-895 was determined by microequilibrium dialysis, as described (31), in mouse plasma (range, 0.32%–1.40%) and human plasma (range, 0.24%–0.60%) and was found to be independent of the concentration in a range of 0.1 to 5 μmol/L.

### CYP3A4-mediated metabolism assays

Human CYP3A4 reactions were performed in mixtures of 100 μL containing TL-895 or the prototypical substrate triazolam (each at 10 μmol/L), CYP3A4 Supersomes (Corning Inc.) at a concentration of 40 pmol/mL, 100 mmol/L potassium phosphate buffer, 3 mmol/L magnesium dichloride, 100 µmol/L β-NADP^+^, 500 μmol/L glucose 6-phosphate, 0.1 U/mL glucose-6-phosphate dehydrogenase, and purified water. Reactions involved incubation at 37°C with constant shaking and were terminated by combining 20 μL of sample with an equal volume of methanol. Samples were collected at time zero and at 0.5, 1, 2, and 4 hours after the start of the reaction and stored at −80°C until analyzed by LC/MS-MS. Details of the analytic method for TL-895 are described below, and triazolam concentrations were determined using a previously described method (32).

### *In vivo* pharmacokinetic studies

All experiments were carried out with age-matched male and female mice on a C57BL/6 (IMSR_JAX:000664) or NOD/SCID gamma (NSG) mouse background strain (NOD.Cg-*Prkdc*^*scid*^*Il2rg*^*tm1Wjl*^/SzJ; RRID: IMSR_JAX:005557). Comparative studies in mice with a deletion of all CYP3A isoforms [CYP3A(−/−); RRID:IMSR_TAC:9047] and wild-type controls were performed in age- and sex-matched mice on an FVB/NTac background strain (FVB.129P2-*Cyp3a13*^*tm1Ahs*^ Del(5Cyp3a57-Cyp3a59)1Ahs; RRID:IMSR_TAC:FVB). Mice weighed between 20 and 30 g at the time of experimentation and were 8 to 14 weeks of age. All studies were approved by the University Laboratory Animal Resources Animal Care and Use Committee at the Ohio State University under approved protocol 2015A00000094-R3. Animals were housed in a temperature- and light-controlled (12-hour light/dark cycles) environment with free access to water and a standard chow diet. A pharmaceutical formulation of TL-895 for administration to mice was prepared by mixing the agent with hydroxypropyl methylcellulose in water (0.25%) to create a 0.6 or 2 mg/mL suspension. Gilteritinib (CID: 49803313) was added to a 0.5% methylcellulose solution in water to create a 6 mg/mL suspension, pravastatin (CID: 16759173) was formulated in sterile saline, and rifampin was dissolved in 10% DMSO in PBS. In all *in vivo* experiments, drugs were administered orally at a TL-895 dose of 3 or 10 mg/kg, a gilteritinib dose of 30 mg/kg, a pravastatin dose of 20 mg/kg, a rifampin dose of 20 mg/kg, or their respective vehicles.

Pharmacokinetic studies were performed as previously described (24). To assess TL-895 plasma concentrations, whole blood samples (30 μL each) were collected from each mouse at 5 (or 15) and 15 (or 30) minutes and at 1, 2, 4, and 8 hours after TL-895 administration. Samples for gilteritinib pharmacokinetic studies were collected at 30 minutes and 1, 3, 6, and 12 hours after administration, and pravastatin samples were collected at 2, 6, 15, 30, and 60 minutes after administration. Studies involving measurement of the endogenous compound chenodeoxycholic acid 24-acyl-β-D-glucuronide (CDCA-24G) were conducted by collecting whole blood samples at baseline (about 30 minutes before drug administration) and at 5 and 15 minutes and 1, 2, and 4 hours after drug administration. When coadministering drugs, the alleged perpetrator drug was administered 30 minutes before the victim drug. In all blood collection schemes, the first three samples were collected from a submandibular vein using a sterile 4 mm Goldenrod animal lancet and a heparinized capillary tube, and the fourth, fifth, and sixth samples were collected from the retro-orbital venous plexus using capillary tubes after the mice were anesthetized with 2% isoflurane. Whole blood samples were centrifuged at 1,500 × *g* for 5 minutes, and the plasma supernatant was collected, immediately placed on dry ice, and subsequently stored at −80°C until analysis by LC/MS-MS.

### Bioanalytical methods

The quantification of TL-895 was performed using a Vanquish ultrahigh-performance liquid chromatography system coupled with a Quantiva triple quadrupole mass spectrometer (Thermo Fisher Scientific; RRID:SCR_018650). Analytic grade TL-895 with a purity of >99.82% was obtained from Selleckchem, and evobrutinib with a purity of 99.95% (Selleckchem) was used as an internal standard. Separation of the analytes of interest was achieved on an ACQUITY UPLC BEH C18 column (130 Å; 1.7 μm; 2.1 × 50 mm; Waters Co.) with a corresponding VanGuard pre-column (ACQUITY UPLC BEH Shield RP18 pre-column). The column temperature was maintained at 40°C, and the autosampler was maintained at 4°C, with a total run time of 3.0 minutes.

The mass spectrometry conditions were optimized for maximum response using heated electrospray ionization in positive ionization mode. Scheduled selective reaction monitoring was employed to detect and quantify TL-895 (m/z 448.252 → 428.22) using evobrutinib (m/z 430.262 → 98.08) as the internal standard. Calibration curves, generated from eight nonzero calibrators within a concentration range of 0.5 to 100 ng/mL, exhibited adequate linearity (r^2^ > 0.99) over 4 days. Quality control samples were prepared at five concentration levels: 0.5, 1.5, 40, 90, and 900 ng/mL, with the latter samples diluted 10 times with blank mouse plasma before sample preparation. The accuracy, expressed as a percentage of bias, across the five quality control levels ranged from −6.1% to 2.57%. The method was validated in accordance with FDA guidelines for bioanalytical method validation (33).

TL-895 was extracted from mouse plasma using a protein precipitation method. A 10 μL aliquot of plasma was transferred to a 0.5 mL Eppendorf tube, to which 5 μL of an internal standard working solution (5 ng/mL) and 85 μL of acetonitrile were added. The samples were briefly vortexed and then centrifuged at 13,000 rpm for 10 minutes at 4°C. Subsequently, 60 μL of the supernatant was transferred to a WebSeal 96-well plate sealed with a WebSeal mat (Thermo Fisher Scientific). A 5 μL volume of the processed sample was analyzed. Data acquisition and processing were performed using Thermo Scientific Xcalibur software (version 4.4.16.14; RRID:SCR_014593).

Concentrations of CDCA-24G, pravastatin, and gilteritinib in mouse plasma were measured using previously validated LC/MS-MS methods (34, 35). Optimization and validation of the pravastatin bioanalytical method are detailed in the Supplementary Information, Appendix.

### Pharmacokinetic and statistical data analysis

Pharmacokinetic parameters were estimated by noncompartmental analysis using PKanalix software and included the observed C_max_ and the area under the plasma concentration–time curve (AUC). The linear trapezoidal method was used to estimate AUC for concentration–time data points. All data are presented as mean ± SD, unless stated otherwise. Group differences (<2 groups) were assessed for statistical significance using an unpaired Student *t* test. Group differences (>2 groups) were assessed for statistical significance using a one-way ANOVA with Tukey multiple comparison. Group differences >2 groups with independent variables were assessed for statistical significance using a two-way ANOVA with Bonferroni’s correction. All statistical tests were two-tailed, and *P* < 0.05 was considered statistically significant across all the studies. Statistical tests were conducted via GraphPad Prism 10.0.2 (GraphPad Software; RRID:SCR_002798).

### Data availability

The datasets used are available from the corresponding author upon reasonable request.

## Results

### Kinase selectivity, potency, and intracellular binding of TL-895

To evaluate the kinase selectivity of TL-895, we utilized a KINOME*scan* against 403 nonmutant kinase targets at a drug concentration of 500 nmol/L and found that TL-895 is highly selective for nonreceptor protein kinases belonging to the TEC family (Fig. 1A), achieving selectivity scores S(35) of 0.015, S(10) of 0.005, and S(1) of 0.002. The two most potent hits were BTK and the related TEC family member, BMX, with %Ctrl values of 0.3% and 3.9%, respectively, followed by TEC and B-lymphocyte kinase with %Ctrl values of 11% to 12%. To validate BTK and BMX as the lead hits from the KINOME*scan*, we utilized the HotSpot Kinase Assay and found EC_50_ values of TL-895 against BTK and BMX of 3.02 and 0.53 nmol/L, respectively (Fig. 1B). NanoBRET intracellular binding assays in HEK293 cells confirmed that TL-895 retains potency against BTK and BMX under physiologically relevant conditions, with observed EC_50_ values of 6.8 and 1.6 nmol/L, respectively (Fig. 1C; Supplementary Fig. S1). Collectively, these results confirm that TL-895 is a highly selective and potent inhibitor of TEC family kinases, especially BTK and BMX.

### Interaction of TL-895 with OATP1B-type transporters

**Figure 2.**
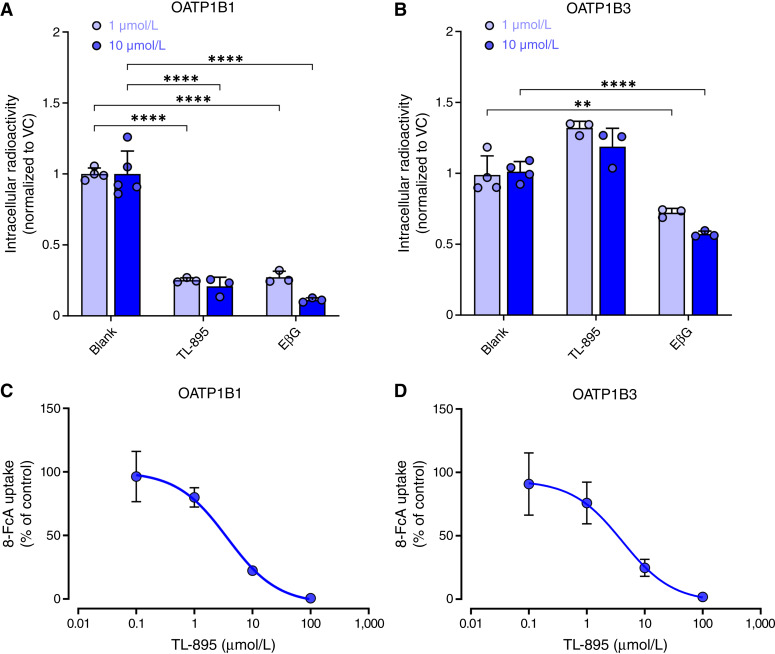
*In vitro* transport and transport inhibition by TL-895. Competitive counterflow of [^3^H]-EβG efflux in the presence or absence of TL-895 or unlabeled EβG in HEK293 cells overexpressing (**A**) OATP1B1 or (**B**) OATP1B3. Data represent mean values (bars) and SD (error bars) representative of four independent experiments performed in triplicate. Concentration-dependent inhibition of 8-FcA transport by TL-895 in HEK293 cells overexpressing (**C**) OATP1B1 or (**D**) OATP1B3. Data represent the mean values (symbols) and SD (error bars) of two independent experiments performed in triplicate. A two-way ANOVA was used to compare the main effects of concentration and treatment group on normalized intracellular radioactivity. **, *P* < 0.01 and ****, *P* < 0.0001.

Due to the important role of OATP1B1 and OATP1B3, two partially redundant organic anion transporting polypeptides expressed on the basolateral membrane of hepatocytes, in the hepatic uptake of xenobiotics (36), and given their sensitivity to TKI-mediated inhibition (37), we sought to characterize the potential interaction of TL-895 with these two transporters. Competitive counterflow assays with TL-895 and OATP1B1 (Fig. 2A) and OATP1B3 (Fig. 2B) in genetically engineered HEK293 cells revealed that TL-895 induces EβG efflux after TL-895 uptake by OATP1B1 but not by OATP1B3, implying that the agent is likely itself a transported substrate of OATP1B1. Ensuing cellular uptake assays revealed that TL-895, compared with other TKIs (37), exhibits relatively weak inhibitory properties against both OATP1B1 (IC_50_, 3.67 μmol/L; Fig. 2C) and OATP1B3 (IC_50_, 4.09 μmol/L; Fig. 2D) when using 8-FcA as the test substrate. Similar results were obtained using EβG as the test substrate for OATP1B1 and CCK-8 as the test substrate for OATP1B3 (Supplementary Fig. S2), implying that the observed inhibitory potential against these transporters is substrate independent.

### Interaction of TL-895 with CYP3A4

**Figure 3.**
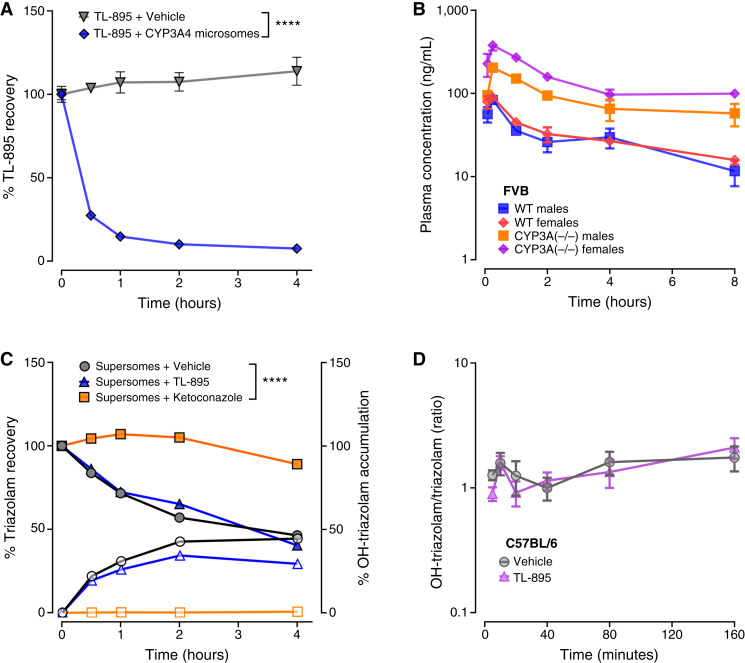
Interaction of TL-895 with CYP3A4 and CYP3A4-mediated metabolism. Metabolism of TL-895 by CYP3A4 microsomes (**A**) and the influence of CYP3A deficiency [CYP(−/−)] on the circulating concentration of oral (3 mg/kg) TL-895 in mice (**B**; *N* = 5 per group). Influence of TL-895 (10 μmol/L) and the positive control inhibitor ketoconazole (10 μmol/L) on the disappearance of the CYP3A4 substrate triazolam (left *Y*-axis) and the formation of its metabolite OH-triazolam (right *Y*-axis) in the presence of CYP3A4 microsomes (**C**). *In vivo* ratio of OH-triazolam to parent triazolam recovery in C57BL/6 male mice with vehicle or TL-895 10 mg/kg oral administration (**D**; *N* = 6 per group). Microsome data represent mean values (symbols) and SD (error bars, if larger than the symbol) of experiments performed in triplicate. In vivo data represent mean values (symbols) and SE (error bars). A two-way ANOVA was used to compare the main effects of time and treatment group on the percentages of TL-895, triazolam, and OH-triazolam recovery. ****, *P* < 0.0001. WT, wild type.

As most FDA-approved TKIs are predominantly metabolized by CYP3A4 (38), we next sought to assess the metabolism of TL-895 by this enzyme to further characterize its potential DDI liability. The incubation of TL-895 with CYP3A4 human Supersomes resulted in an almost complete loss of the parent compound within 2 hours, implying that TL-895 undergoes extensive CYP3A4-mediated metabolism (Fig. 3A). Ensuing studies involving CYP3A(−/−) mice, which lack all murine orthologs of human CYP3A4, indicated that the systemic exposure to TL-895, as measured by changes in AUC, was increased by 1.9-fold (*P* < 0.001) in male mice and by 4.6-fold (*P* < 0.01) in female mice compared with age-matched wild-type counterparts (Fig. 3B; Table 1). Interestingly, TL-895 did not substantially inhibit the function of CYP3A4, as evaluated using CYP3A4-mediated triazolam hydroxylation, in comparison with the known CYP3A4 inhibitor, ketoconazole (Fig. 3C). Consequent studies of male C57BL/6 mice with TL-895 or vehicle revealed a lack of CYP3A inhibition as indicated by the near-equivalent ratio of OH-triazolam metabolite formation to parent triazolam recovery (Fig. 3D). Although the mechanistic basis for the sexually dimorphic effect of CYP3A deficiency on the pharmacokinetic profile of TL-895 is unclear, it is noteworthy that plasma levels tended to be slightly higher in female mice than in male mice on a C57BL/6 wild-type (Supplementary Fig. S3A) or NSG wild-type background strains (Supplementary Fig. S3B). However, the sexually dimorphic effect in TL-895 pharmacokinetics was not consistent across exposure metrics, the dose administered, and the background strain of the mice (Table 1), implying that CYP3A may not be the primary determinant of sexual dimorphic pharmacokinetics for TL-895. Although elucidating sexual dimorphism is beyond the scope of the present work, the tendency toward a larger effect size in the pharmacokinetics of TL-895 in female mice may be attributed to gastrointestinal differences in gastric emptying or intestinal transit time that can affect the extent of drug absorption in rodents (39, 40) or differential expression in uptake and efflux transporters that contribute to TL-895 disposition and elimination (41, 42).

**Table 1:** Dependence of TL-895 pharmacokinetics on mouse strain, CYP3A genotype, TL-895 dose, and sex

Strain or genotype	TL-895 dose (mg/kg)	Sex	C_max_ (ng/mL)	AUC (ng × h/mL)
C57BL/6 WT	10	Male	276 ± 36.5	693 ± 94.2
		Female	509 ± 62.2****	869 ± 75.0**
	3	Male	122 ± 31.6	95.7 ± 9.55
		Female	237 ± 96.3*	110 ± 49.7
NSG WT	10	Male	254 ± 28.6	681 ± 55.8
		Female	279 ± 37.1	1,220 ± 86.9****
	3	Male	190 ± 32.3	395 ± 131
		Female	176 ± 19.3	414 ± 17.5
FVB WT	3	Male	96.7 ± 9.15	318 ± 40.0
		Female	89.3 ± 10.0	246 ± 16.1**
FVB CYP3A(−/−) (3 mg/kg)		Male	181 ± 13.6^####^	571 ± 72.1^###^
		Female	345 ± 49.8***^, ####^	1,130 ± 78.8****^, ####^

Data values presented represent the mean ± SD (*N* = 5 per condition).

*P* values presented represent the unpaired Student *t* test comparison between genders (*) or CYP3A genotype (#). *, *P* < 0.05; **, *P* < 0.01; ***, ^###^, *P* < 0.001; and ****, ^####^, *P* < 0.0001.

Abbreviations: AUC, AUC from time 0 to the last time point with measurable levels of TL-895; WT, wild type.

### *In vivo* DDI liabilities with TL-895 via OATP1B or CYP3A inhibition

To further explore the ability of TL-895 to cause DDIs mediated via inhibition of OATP1B-type transporters (affecting hepatic uptake) and/or CYP3A-type enzymes (affecting hepatic metabolism), *in vivo* studies with TL-895 were performed evaluating effects on CDCA-24G, an endogenous biomarker of OATP1B-type transport function (43); pravastatin, a xenobiotic probe substrate of OATP1B1 (44); and gilteritinib, an antileukemic substrate of both OATP1B1 and CYP3A4 (45). Compared with pretreatment with the positive control OATP1B1 inhibitor, rifampin, we observed that TL-895 did not influence circulating concentrations of CDCA-24G (Supplementary Fig. S4A) or pravastatin (Supplementary Fig. S4B). Moreover, pretreatment with TL-895 did not significantly alter the pharmacokinetic profile of gilteritinib in male or female mice on a C57BL/6 (Supplementary Fig. S4C) or NSG background strain (Supplementary Fig. S4D). These collective observations are consistent with our preclinical *in vitro* predictions and imply that the likelihood of TL-895 causing OATP1B1- or CYP3A4-mediated DDIs is low (Table 2).

**Table 2:** Pharmacokinetic DDIs induced by TL-895 in mice

Strain	Victim (dose in mg/kg)	Sex	Perpetrator (dose in mg/kg)	C_max_ (ng/mL)	AUC (ng × h/mL)
C57BL/6	CDCA-24G	Male	Vehicle	19.5 ± 9.77	46.0 ± 22
			Rifampin (20)	39.8 ± 6.65^##^	112 ± 21^###^
			TL-895 (10)	14.7 ± 3.37	34.0 ± 13
	Pravastatin (20)	Male	Vehicle	406 ± 186	102 ± 42
			Rifampin (20)	994 ± 148^####^	333 ± 64^###^
			TL-895 (10)	182 ± 31	49.8 ± 7.60
	Gilteritinib (30)	Male	Vehicle	469 ± 51	4,960 ± 459
		Female		787 ± 102***	6,980 ± 1,090**
		Male	TL-895 (3)	502 ± 113	5,130 ± 661
		Female		688 ± 97*	6,440 ± 603*
NSG	Gilteritinib (30)	Male	Vehicle	440 ± 86	4,410 ± 406
		Female		593 ± 87*	4,930 ± 581
		Male	TL-895 (3)	421 ± 20	4,370 ± 436
		Female		507 ± 28***	4,830 ± 661

Data values presented represent the mean ± SD (*N* = 4–5 per condition).

*P* values presented represent an unpaired Student *t* test on sex (*) or an ordinary one-way ANOVA with Tukey multiple comparisons test on perpetrator drug versus vehicle (#). *, *P* < 0.05; **, ^##^, *P* < 0.01; ***, ^###^, *P* < 0.001; and ****, ^####^, *P* < 0.0001. TL-895 had no statistically significant effect on CDCA-24G or pravastatin.

## Discussion

In the present study, we characterized the pharmacokinetic profile of TL-895, an investigational, second-generation BTK inhibitor for the treatment of B-cell malignancies. First, we validated that TL-895 is a highly selective inhibitor of BTK and BMX, supporting its potential to overcome off-target toxicities or resistance induced by current TEC family kinase inhibitors (12–14). Based on the importance of CYP3A (38) and OATP1B-type transport (22) to the disposition of TKIs, including competing BTK inhibitors (21), we investigated the potential for TL-895 to act as a perpetrator or victim in pharmacokinetic DDIs involving OATP1B-type transport and CYP3A4-mediated metabolism. These studies utilized robust methodology and novel tools that our lab has previously used to characterize the importance of CYP3A and OATP1B in the disposition of venetoclax (29), gilteritinib (45), and ibrutinib (46). In the present studies, we identified TL-895 as a substrate of both OATP1B1 and CYP3A4. These studies shed light on the mechanisms involved in the elimination of TL-895 and have potential implications for the design of future DDI studies involving perpetrator or victim drugs that are dual substrates of OATP1B-mediated transport and CYP3A-mediated metabolism.

We focused initially on DDI liabilities in which TL-895 may act as a perpetrator in a manner that is dependent on the inhibition of the two human OATP1B-type transporters, OATP1B1 and OATP1B3, based on the notion that TL-895 shares structural and pharmacologic features with several TKIs that are known substrates and/or inhibitors of OATP1B-type transporters [e.g., molecular weight, polar groups (47)]. Ensuing *in vitro* studies verified our inhibitor hypothesis by demonstrating that TL-895, in a concentration-dependent manner, inhibited the uptake of various known substrates of OATP1B1 and OATP1B3 (Fig. 2). We previously reported that the ability of some, but not all, TKIs to inhibit OATP1B1 (37) and OATP1B3 (48) is dependent on LYN, a protein kinase that activates these transporters via a posttranslational mechanism involving tyrosine phosphorylation. Interestingly, we found that LYN kinase was insensitive to inhibition by TL-895 (Supplementary Fig. S5) in a relevant range of concentrations, especially in comparison with the known LYN inhibitor, nilotinib (49). This suggests that the inhibitory properties of TL-895 against OATP1B-type transporters may involve an undefined competitive (OATP1B1) and/or allosteric (OATP1B3) mechanism. Importantly, as C_max_/IC_50_ values were ≤0.1 and R values were ≤1.1, it is likely that the inhibition of OATP1B-type transporters by TL-895 occurs only at supraphysiologic concentrations. This supposition is in line with *in vivo* studies conducted in translationally relevant mouse models with doses selected to mimic concentrations previously observed in human patients (2), which indicated that TL-895 does not significantly influence circulating concentrations of the OATP1B1 substrates CDCA-24G, pravastatin, and gilteritinib. Although the single murine ortholog transporter OATP1B2 shares a high degree of sequence homology with the human OATP1B-type transporters as well as similarity in basolateral membrane localization and overlapping substrate specificity (50), it should be pointed out that unlike in humans, mouse hepatocytes express multiple members of OATP1A-type transporters, a subfamily of solute carriers that can potentially provide compensatory restoration of function when OATP1B2 is inhibited (51). Pharmacokinetic studies in OATP1B2-deficient mice and humanized mice expressing both OATP1B1 and OATP1B3 would be required to determine if, in such models, the lack of OATP1B-related DDI phenotypes, as observed in wild-type mice, can be recapitulated (52).

As concentration-dependent inhibition of OATP1B-type transporters is a common feature of transported substrates, we evaluated TL-895 in a competitive counterflow assay, an indirect method commonly used to identify novel substrates (36), and found that TL-895 is itself a substrate of the main human OATP1B-type transporter, OATP1B1. Although a more reliable direct cellular uptake assay overcoming the nonspecific extracellular membrane binding typically observed with TKIs is warranted (22), this finding supports the thesis that uptake carriers capable of transporting TL-895 need to be expressed in the liver so that the drug can be taken up in advance of hepatic metabolism. *In vitro* microsomal studies and *in vivo* experiments using enzyme-deficient mouse models confirmed that hepatic metabolism of TL-895 is extensive and involves the enzyme CYP3A4, an observation that is in line with existing data for many structurally related TKIs (38). Importantly, TL-895 did not inhibit the function of this enzyme or reduce triazolam metabolism, suggesting that the agent is a potential victim of DDIs involving CYP3A4-mediated metabolism but is unlikely to be a perpetrator of such interactions at clinically achievable concentrations.

Despite our comprehensive evaluation of CYP3A and OATP1B-type transporters in the disposition of TL-895, additional DDI liabilities will need to be assessed in future work. In particular, the impact of efflux transport through ABC transporters, including P-gp, should be evaluated, especially considering that substrates of CYP3A are often also substrates for P-gp (53). Indeed, BTK inhibitors have been found to be both substrates (11, 54) and inhibitors of P-gp (55). However, although CYP3A deficiency has resulted in dramatic changes in the oral bioavailability of the BTK inhibitor ibrutinib (46), P-gp deficiency had only minimal effects on overall exposure (56). These data potentially imply that DDI affecting hepatic clearance, which is typically rate-limited by CYP3A and hepatic OATPs (57), may have a larger impact on exposure compared with those affecting P-gp and absorption. Nonetheless, P-gp transport is an important determinant of organ accumulation and brain penetration (56) and remains an important consideration for the development of TL-895.

While characterizing DDI liabilities, we found that TL-895 inhibits the TEC kinase, BMX, more potently than its presumed primary target, BTK. This observation is of potential translational relevance, as we previously reported that BMX is implicated in resistance to gilteritinib in *FLT3*-mutated acute myeloid leukemia, and this provides justification for further investigations into the use of TL-895 as a BMX inhibitor in this indication (58–60). In particular, we recently found that BMX can activate alternative signaling pathways via modulation of the cytokine and chemokine network, thereby promoting drug resistance through bone marrow microenvironmental support, and disruption of this support may improve the clinical outcomes with gilteritinib-containing regimens. Indeed, the reversal of this resistance mechanism by TL-895 could potentially improve the outcome of treatment, and such a strategy could be explored with the knowledge that the pharmacokinetic profile of gilteritinib, a substrate of both OATP1B-type transporters and CYP3A4 (45), is not negatively influenced by TL-895.

Collectively, our findings signify that OATP1B1 and CYP3A4 contribute to the *in vivo* handling of the dual BTK/BMX inhibitor TL-895 although the agent is an unlikely perpetrator of potentially deleterious DDIs in polypharmacy regimens. We anticipate that the experimental template outlined here for TL-895 is applicable to other investigational small-molecule therapeutics and could be exploited to predict DDI liabilities that may have clinical significance. Applying this pipeline to understanding the DDI liability of developmental therapeutics will prevent costly and harmful DDIs by informing appropriate dose adjustments and combinatorial strategies.

## Supplementary Material

Supplementary Figure S1 Positive control inhibition of BTK and BMX in NanoBRET assay. BRET luciferase response of BTK and BMX in the presence of CTx-0294885 or dasatinib, respectively, in HEK293 cells expressing BTK- and BMX-NanoLuc Fusion Vector showing EC50 values of 30.1 nM and 4.0 nM, respectively.

Supplementary Figure S2 Inhibition of OATP1B1 and OATP1B3 by TL-895. Inhibition of EβG transport by OATP1B1 (A) and CCK-8 transport by OATP1B3 (B) in the presence and absence of Varying concentrations of TL-895 in engineered HEK293 cells. Data represent mean values (symbols) and SD (error bars) of 2 independent experiments performed in triplicate.

Supplementary Figure S3 Pharmacokinetic profile of TL-895 in C57BL/6 and NSG mice. TL-895 plasma concentrations were determined in male and female mice on a C57BL/6 (A) or NSG (B) background strain. Mice received a single oral dose of 3 or 10 mg/kg. Data represent mean values (symbols) and SD (error bars) using 5 animals per group.

Supplementary Figure S4 Assessment of DDI liabilities induced by TL-895. Influence of TL-895 (3 mg/kg) on circulating concentrations of CDCA-24G (A), oral (20 mg/kg) pravastatin (B), or oral (30 mg/kg) gilteritinib in C57BL/6 mice (C) and NSG mice (D). Data represent mean values (symbols) and SD (error bars) using 4 or 5 animals per group.

Supplementary Figure S5 Activity of TL-895 on LYN kinase. KINOMEscan activity of TL-895 and nilotinib, a positive control, against LYN kinase, a post-translational regulator of OATP1B transporter activity. Data represent mean values (symbols) and SD (error bars) of 2 biological replicate experiments.

Supplementary Methods Supplementary Methods
