# Supplementary figures and images for: Drug–Drug Interaction Liabilities with BTK Inhibitor TL-895
Source: Cancer Res Commun. 2025 Sep 12;5(9):1621–30. doi: 10.1158/2767-9764.CRC-25-0265 (PMC12426595; doi:10.1158/2767-9764.CRC-25-0265)

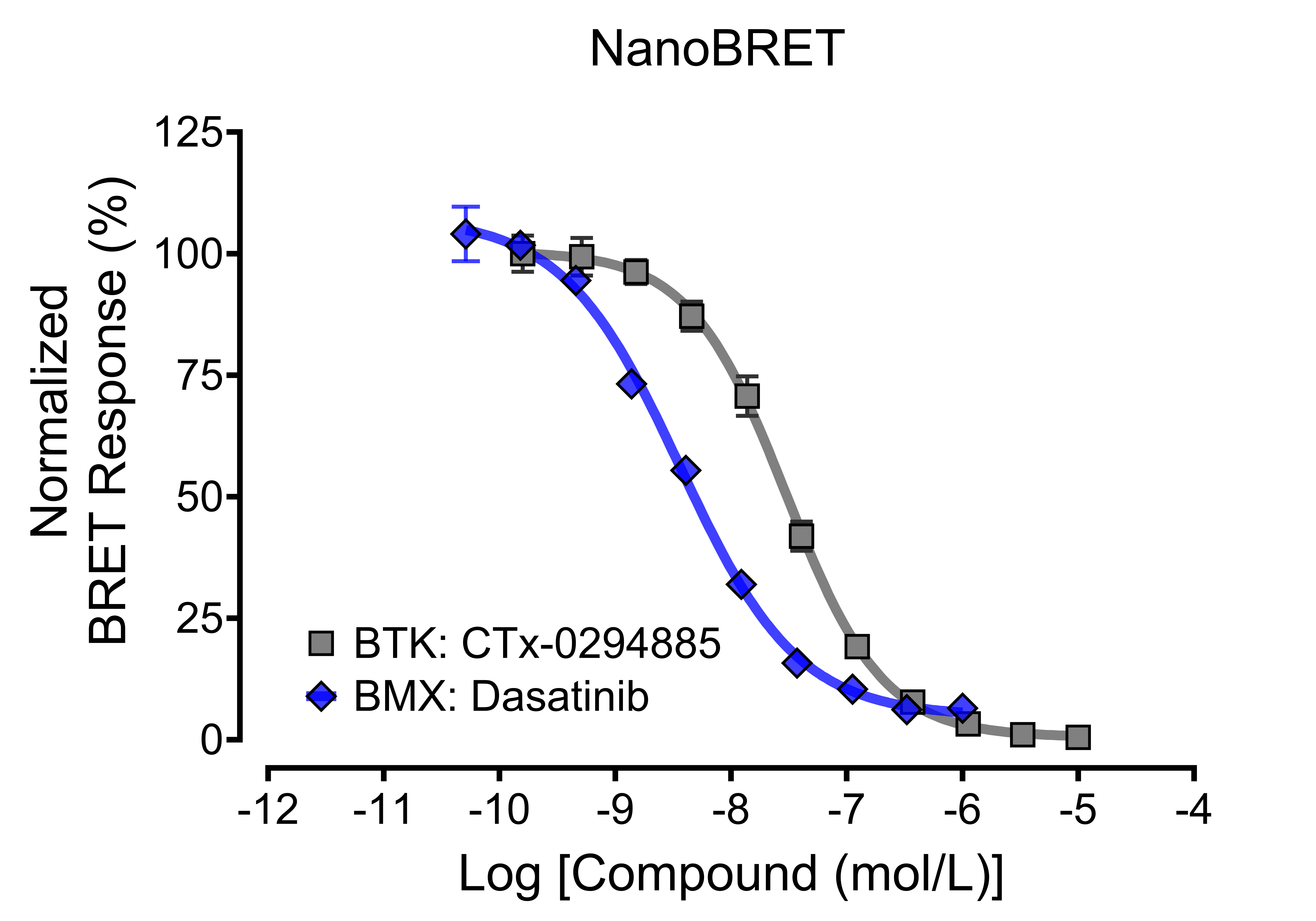

Supplement: Supplementary Figure S1 — Positive control inhibition of BTK and BMX in NanoBRET assay. BRET luciferase response of BTK and BMX in the presence of CTx-0294885 or dasatinib, respectively, in HEK293 cells expressing BTK- and BMX-NanoLuc Fusion Vector showing EC50 values of 30.1 nM and 4.0 nM, respectively. [file crc-25-0265_supplementary_figure_s1_supps1.png]

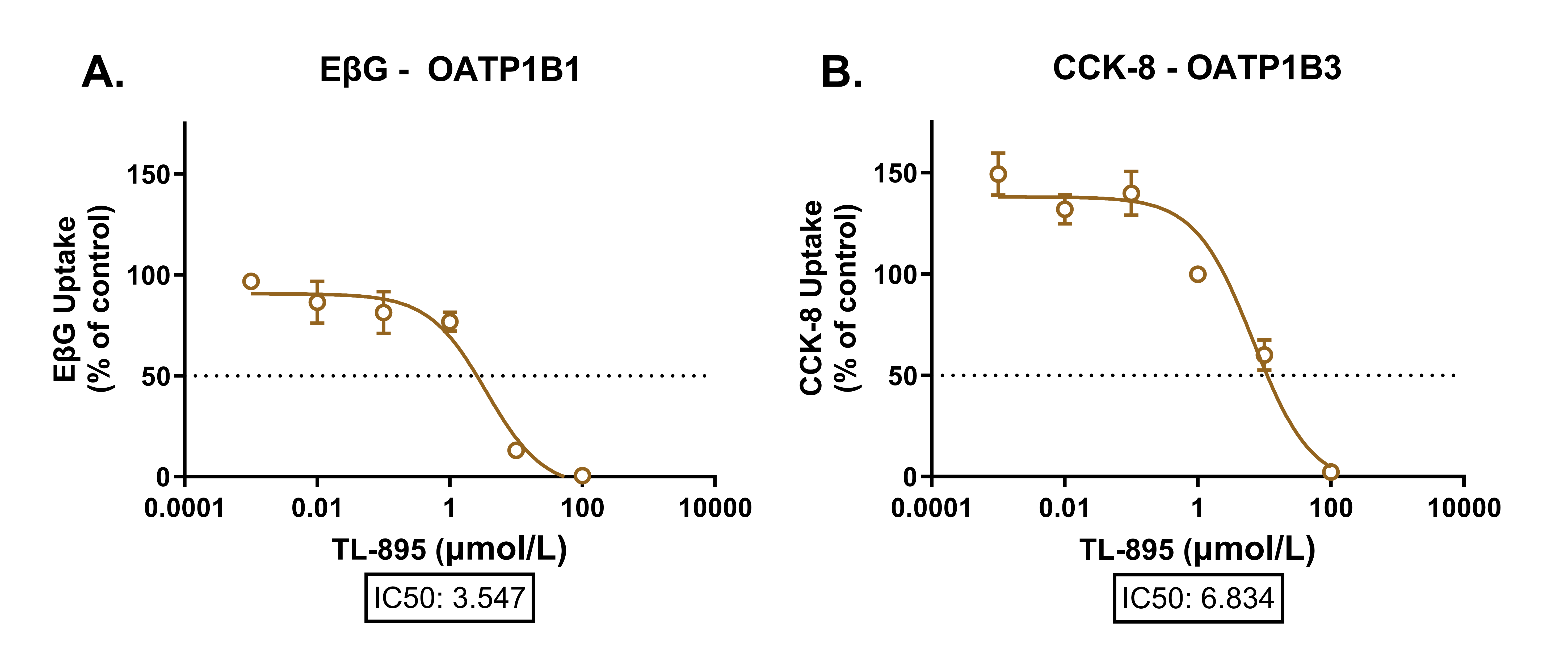

Supplement: Supplementary Figure S2 — Inhibition of OATP1B1 and OATP1B3 by TL-895. Inhibition of EβG transport by OATP1B1 (A) and CCK-8 transport by OATP1B3 (B) in the presence and absence of Varying concentrations of TL-895 in engineered HEK293 cells. Data represent mean values (symbols) and SD (error bars) of 2 independent experiments performed in triplicate. [file crc-25-0265_supplementary_figure_s2_supps2.png]

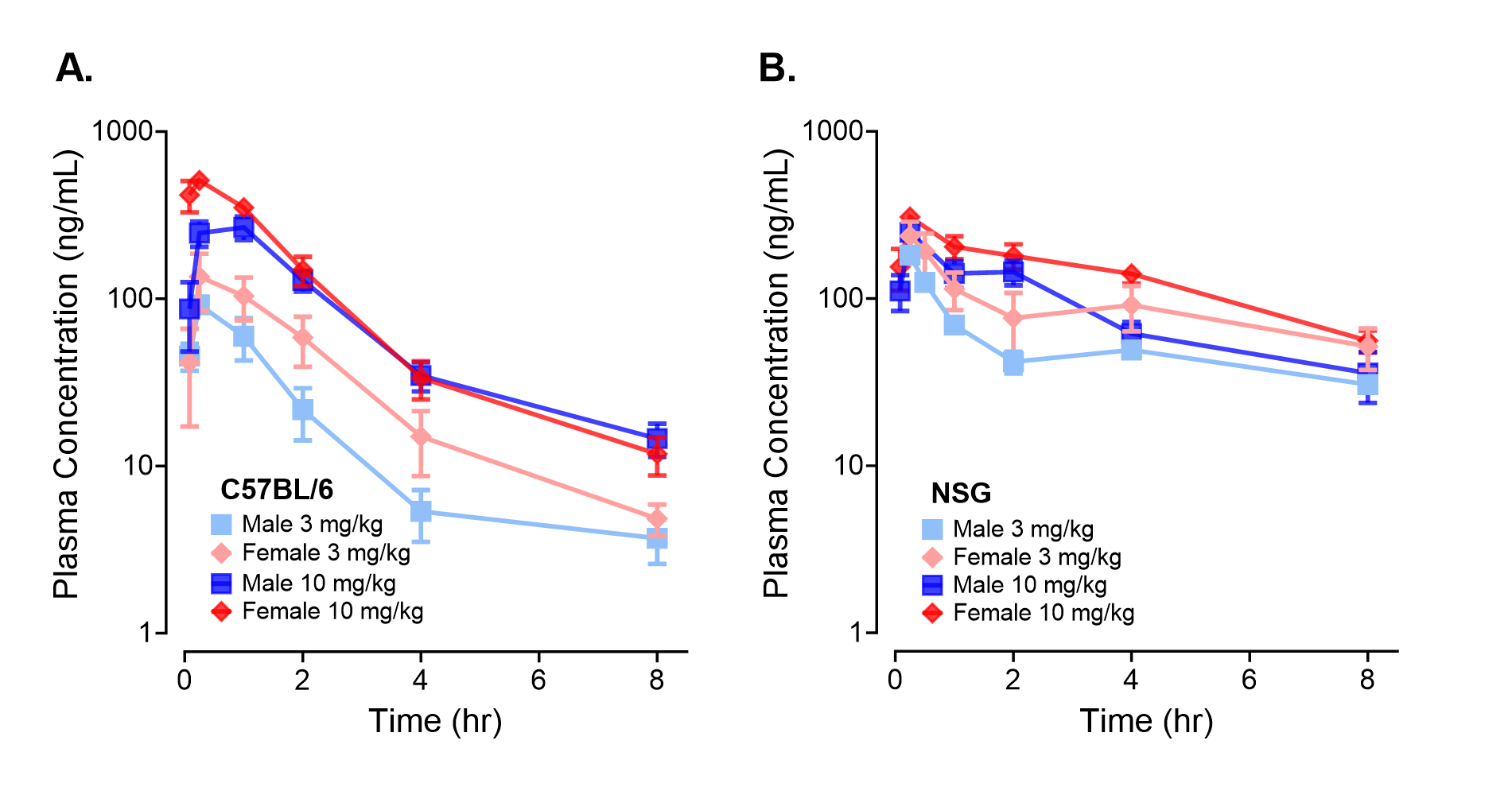

Supplement: Supplementary Figure S3 — Pharmacokinetic profile of TL-895 in C57BL/6 and NSG mice. TL-895 plasma concentrations were determined in male and female mice on a C57BL/6 (A) or NSG (B) background strain. Mice received a single oral dose of 3 or 10 mg/kg. Data represent mean values (symbols) and SD (error bars) using 5 animals per group. [file crc-25-0265_supplementary_figure_s3_supps3.png]

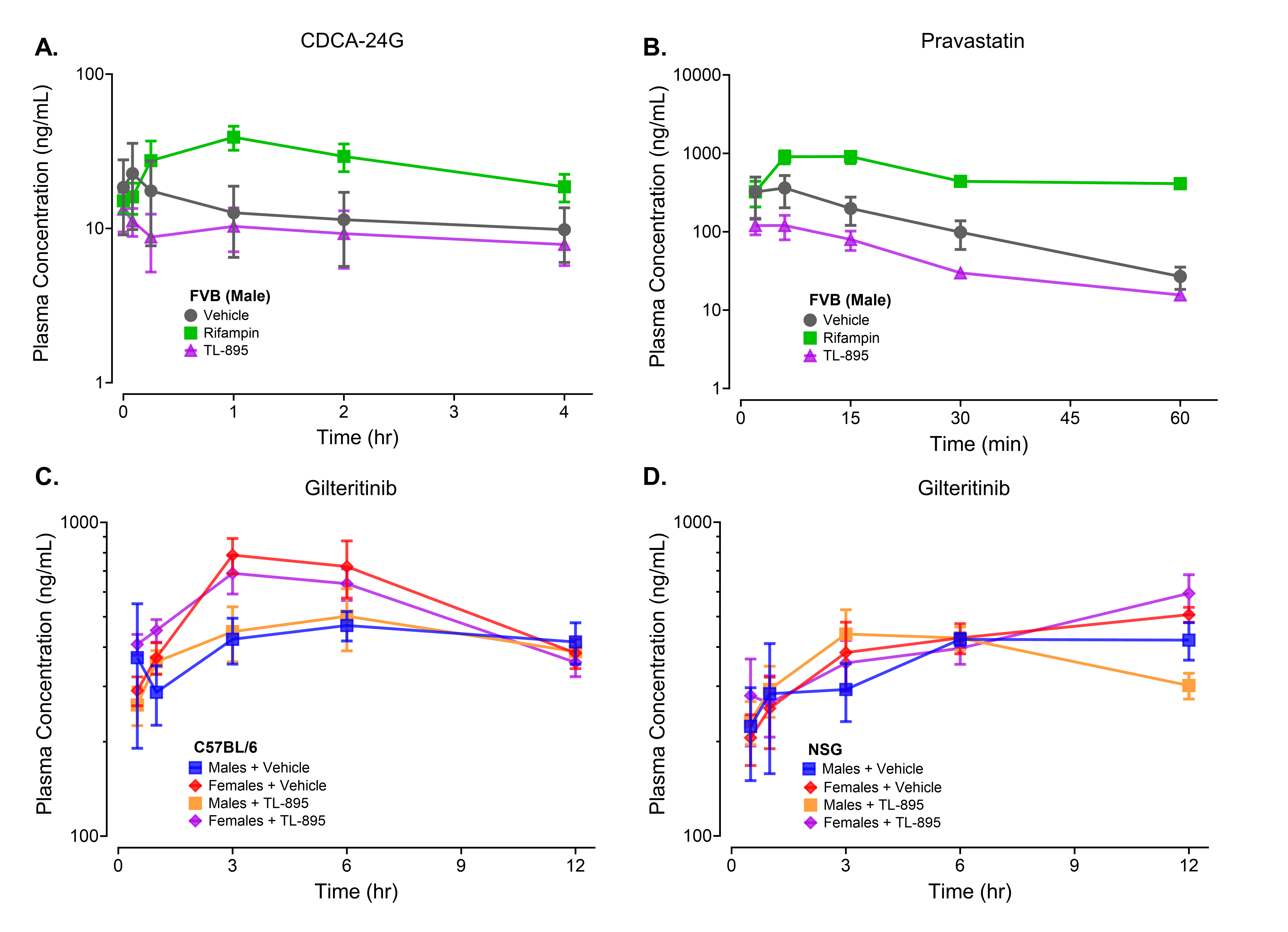

Supplement: Supplementary Figure S4 — Assessment of DDI liabilities induced by TL-895. Influence of TL-895 (3 mg/kg) on circulating concentrations of CDCA-24G (A), oral (20 mg/kg) pravastatin (B), or oral (30 mg/kg) gilteritinib in C57BL/6 mice (C) and NSG mice (D). Data represent mean values (symbols) and SD (error bars) using 4 or 5 animals per group. [file crc-25-0265_supplementary_figure_s4_supps4.png]

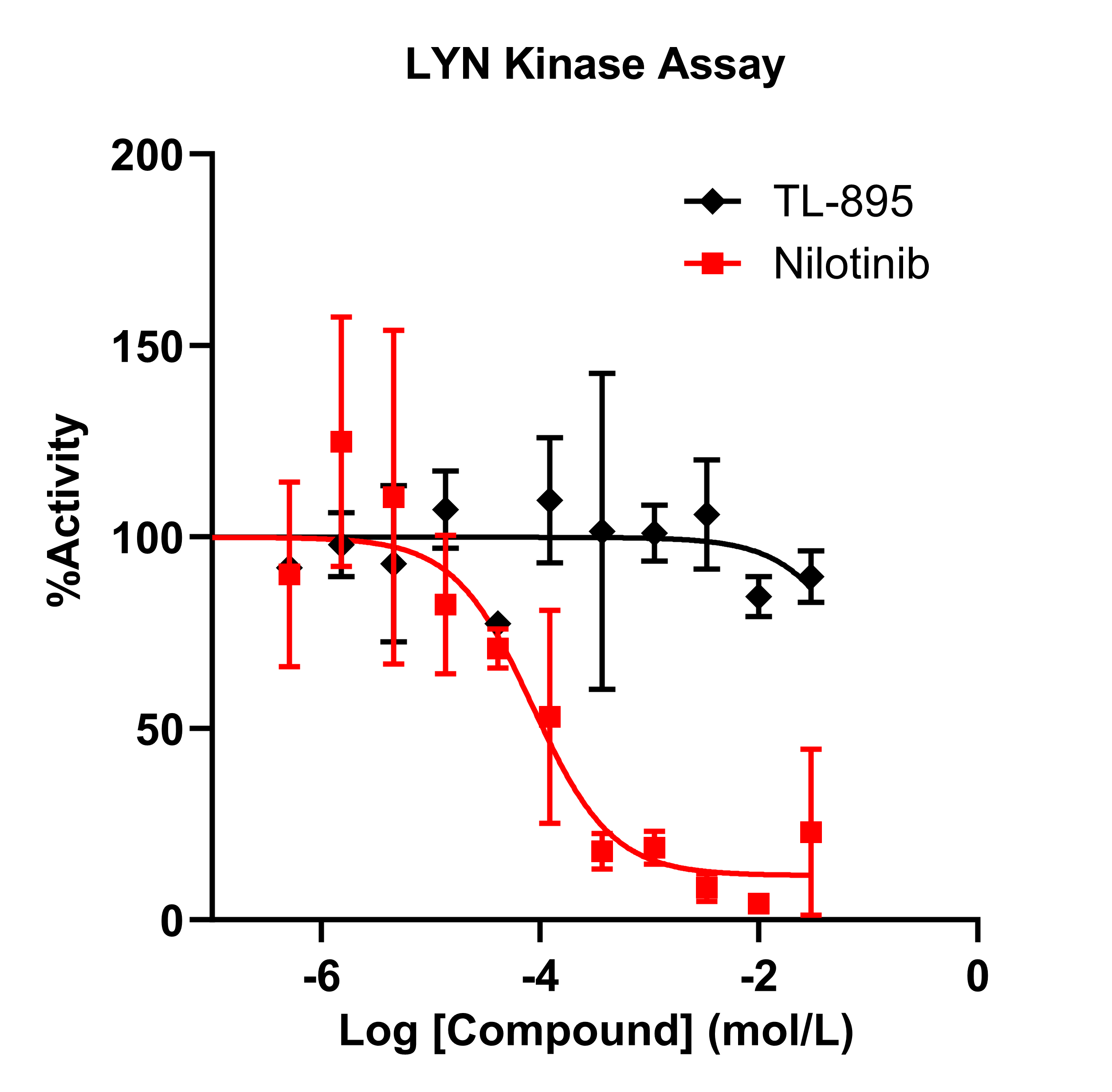

Supplement: Supplementary Figure S5 — Activity of TL-895 on LYN kinase. KINOMEscan activity of TL-895 and nilotinib, a positive control, against LYN kinase, a post-translational regulator of OATP1B transporter activity. Data represent mean values (symbols) and SD (error bars) of 2 biological replicate experiments. [file crc-25-0265_supplementary_figure_s5_supps5.png]
