# Supplementary material for: Drug–Drug Interaction Liabilities with BTK Inhibitor TL-895
Source: Cancer Res Commun. 2025 Sep 12;5(9):1621–30. doi: 10.1158/2767-9764.CRC-25-0265 (PMC12426595; doi:10.1158/2767-9764.CRC-25-0265)
Supplement: Supplementary Methods [file crc-25-0265_supplementary_methods_suppsm.docx]

**Supplementary Information**

**Exploration of drug-drug interaction liabilities induced by the BTK inhibitor TL-895**

Jack C. Stromatt, Eman A. Ahmed, Thomas Drabison, Mahesh Nepal, Anika Chowdury, Shelley J. Orwick, Daelynn R. Buelow, Eric D. Eisenmann, Kevin M. Huang, Alex Sparreboom, and Sharyn D. Baker

**Supplementary Materials and Methods**

**Bioanalytical method for pravastatin**

A Vanquish Ultra-High-Performance Liquid Chromatography system coupled with an Altis triple quadrupole mass spectrometer (Thermo Fisher Scientific) was utilized for quantification of pravastatin (Purity 99.94%; Selleckchem) and its internal standard, pravastatin-d3 (purity >99.9%; Cayman Chemical). Chromatographic separation was performed on an Accucore aQ column (80 Å; 2.6 µm; 2.1 × 50 mm; Thermo Fisher Scientific) equipped with a C18 AQUASIL guard cartridge (3 µm; 2.1 × 10 mm; Thermo Fisher Scientific). The column was held at a temperature of 40°C, while the autosampler rack was maintained at a temperature of 4°C. The mobile phase consisted of 0.1% formic acid in LC-MS grade water (solvent A) and a mixture of 0.1% formic acid in acetonitrile and methanol (1:3) as solvent B, with a total run time of 5.0 min.

Mass spectrometry conditions were optimized to ensure maximum stable response for the analyte and internal standard by infusing a standard mixture of pravastatin and pravastatin-d3 in acetonitrile at a concentration of 0.5 μg/mL using negative ionization mode. Heated electrospray ionization (ESI) served as the ion source, and selective reaction monitoring (SRM) was applied with optimized SRM transitions and collision energies. Detection and quantification of pravastatin (m/z 423.38 → 321.22) were performed using pravastatin-d3 (m/z 426.47 → 321.22) as the internal standard. A diverter valve was employed to minimize impurities, directing the flow to the mass spectrometer only between 1.0 and 4.0 min.

A nine-point calibration curve ranging from 1.25 to 500 ng/mL was constructed, with quality control (QC) samples prepared at five concentration levels, including 1.25, 5, 200, 450, and 4500 ng/mL, respectively, of which the latter QC samples were diluted tenfold in blank mouse plasma before analysis. Data acquisition and processing were performed using Thermo Scientific Xcalibur software (version 4.4.16.14). Calibration curves, generated automatically by Xcalibur, displayed adequate linearity (r² > 0.99) over four days of analysis. The accuracy, expressed as a percent bias, for the QC samples representing the lower limit of quantitation samples was 7.3%, while for other QC samples the accuracy ranged between -5.7% and 8.1%.

Pravastatin was extracted from mouse plasma via protein precipitation, where a 10-μL plasma sample was combined with 5 μL of internal standard solution (10 μg/mL) and 85 μL of methanol. Following vortex mixing and centrifugation at 13,000 rpm for 10 min at 4°C, 60 μL of the supernatant were transferred to a 96-well plate, sealed, and 5-μL aliquots were analyzed.
